# Reducing Medication Costs to Prevent Cardiovascular Disease: A Community Guide Systematic Review

**DOI:** 10.5888/pcd12.150242

**Published:** 2015-11-25

**Authors:** Gibril J. Njie, Ramona K.C. Finnie, Sushama D. Acharya, Verughese Jacob, Krista K. Proia, David P. Hopkins, Nicolaas P. Pronk, Ron Z. Goetzel, Thomas E. Kottke, Kimberly J. Rask, Daniel T. Lackland, Lynne T. Braun, the Community Preventive Services Task Force

**Affiliations:** Author Affiliations: Gibril J. Njie, Ramona K.C. Finnie, Sushama D. Acharya, Verughese Jacob, Krista K. Proia, David P. Hopkins, Centers for Disease Control and Prevention, Atlanta, Georgia; Nicolaas P. Pronk, Thomas E. Kottke, HealthPartners Institute for Education and Research, Minneapolis, Minnesota; Ron Z. Goetzel, Johns Hopkins University, Baltimore, Maryland; Kimberly J. Rask, Emory University, Atlanta, Georgia; Daniel T. Lackland, Medical University of South Carolina, Charleston, South Carolina; Lynne T. Braun, Rush University, Chicago, Illinois.

## Abstract

**Introduction:**

Hypertension and hyperlipidemia are major cardiovascular disease risk factors. To modify them, patients often need to adopt healthier lifestyles and adhere to prescribed medications. However, patients’ adherence to recommended treatments has been suboptimal. Reducing out-of-pocket costs (ROPC) to patients may improve medication adherence and consequently improve health outcomes. This Community Guide systematic review examined the effectiveness of ROPC for medications prescribed for patients with hypertension and hyperlipidemia.

**Methods:**

We assessed effectiveness and economics of ROPC for medications to treat hypertension, hyperlipidemia, or both. Per Community Guide review methods, reviewers identified, evaluated, and summarized available evidence published from January 1980 through July 2015.

**Results:**

Eighteen studies were included in the analysis. ROPC interventions resulted in increased medication adherence for patients taking blood pressure and cholesterol medications by a median of 3.0 percentage points; proportion achieving 80% adherence to medication increased by 5.1 percentage points. Blood pressure and cholesterol outcomes also improved. Nine studies were included in the economic review, with a median intervention cost of $172 per person per year and a median change in health care cost of −$127 per person per year.

**Conclusion:**

ROPC for medications to treat hypertension and hyperlipidemia is effective in increasing medication adherence, and, thus, improving blood pressure and cholesterol outcomes. Most ROPC interventions are implemented in combination with evidence-based health care interventions such as team-based care with medication counseling. An overall conclusion about the economics of the intervention could not be reached with the small body of inconsistent cost-benefit evidence.

## Introduction

High blood pressure and high blood cholesterol (hypertension and hyperlipidemia, respectively) are 2 major cardiovascular disease (CVD) risk factors, yet suboptimal treatment of both remains a persistent problem in the United States. On the basis of recent estimates, approximately 31.1 million — or less than half (46.5%) — of those diagnosed with hypertension have it controlled at recommended levels, even though most Americans with hypertension report having a usual source of health care (89.4%) and health insurance (85.2%) (1). Although national guidelines do not define cholesterol treatment goals, based on earlier guidance, 33% of US adults with high low-density lipoprotein (LDL) cholesterol did not have it controlled at recommended levels (2). Improvement of such low control rates is paramount to reducing the prevalence of CVD in the United States. Because of the aging of the US population, the prevalence of all CVD is projected to increase to 40.5% by 2030, and the total economic burden of CVD is estimated to exceed $1 trillion annually (3).

One approach to mitigating the rising burden of CVD is through improved adherence to a regimen of medication, defined as patients taking medications as prescribed (eg, twice daily) and continuing to take a prescribed medication (4). Despite pharmaceutical advances to treat CVD risk factors, medication adherence remains suboptimal (5). Adherence to blood pressure medication reduces hospitalization risk and health care costs (6); similarly, adherence to statins reduces CVD-related illness and death, but the medications remain underused. Adherence rates range from 25% to 40% among older adults (7,8). Cost-related medication nonadherence is a serious problem in the United States, especially among vulnerable populations such as older adults and people who are disabled, uninsured, or underinsured (9,10).

To reduce medication costs, patients often fill fewer prescriptions, split pills, or skip doses, practices that put them at increased risk for adverse health outcomes (11). Removing cost-related barriers by reducing out-of-pocket costs (ROPC) for medications may improve patients’ medication adherence and related health outcomes. This systematic review examined up-to-date evidence on the effectiveness and economics of policies and programs that reduce patient out-of-pocket costs for medications prescribed to treat hypertension, hyperlipidemia, or both. It also assessed the applicability of findings for various US populations and considerations for implementation of ROPC for medications.

For this review, ROPC for patients with hypertension and hyperlipidemia involves program and policy changes that make medications for CVD more affordable. Costs for treatment medications — generic or brand-name — can be reduced by providing new or expanded treatment coverage and lowering or eliminating patient out-of-pocket expenses (eg, copayments, coinsurances, deductibles). ROPC is coordinated through the health care system with preventive services delivered in clinical or nonclinical settings (eg, worksite, community). ROPC can be implemented alone or in combination with additional interventions to enhance patient–provider interaction such as team-based care, medication counseling, and patient education. Program or policy changes can be made by many implementers, including insurance companies, government agencies, and employers.

## Methods

Detailed systematic review methods used by The Community Guide have been published previously (12,13). For this review, a review coordination team was formed, comprising CVD subject matter experts from various agencies, organizations, and academic institutions, together with qualifıed systematic reviewers from the Community Guide Branch at the Centers for Disease Control and Prevention (CDC). The team worked under the oversight of the independent, unpaid, nonfederal Community Preventive Services Task Force. A systematic review of the economic evidence was conducted along with the effectiveness review. Methods for conducting Community Guide systematic economic reviews are available at www.thecommunityguide.org/about/economics.html.

**Figure 1:**
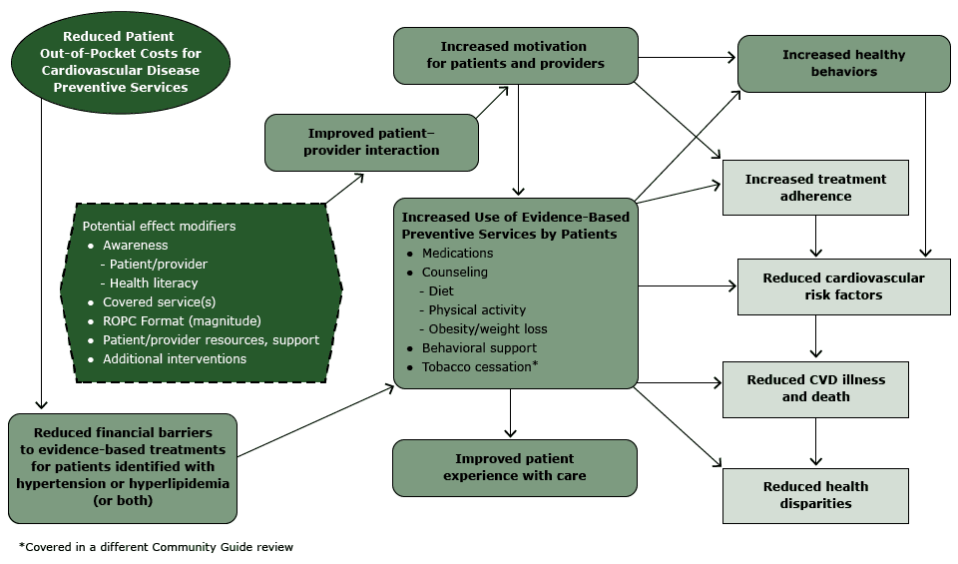
Analytic framework: reduced out-of-pocket costs (ROPC) for cardiovascular disease (CVD) preventive services for patients with hypertension or hyperlipidemia.

The analytic framework (Figure 1) reflects the team’s conceptual approach to evaluating evidence on the effectiveness of ROPC to improve blood pressure and lipid levels. In summary, the team hypothesized that ROPC for patients who have hypertension or hyperlipidemia is likely to reduce financial barriers and thereby increase patient use of CVD preventive services, leading to increased healthy behaviors and treatment adherence, improved patient care experience, and ultimately, reduced CVD risk factors, illness, death, and CVD-related disparities.

The economic rationale for ROPC interventions is developed in a recent article by Baicker and colleagues (14). Copays and coinsurance serve to ensure that the cost to the user is not set so low that services are overused, the well-recognized problem of moral hazard. Less recognized is that price ought not to be set so high that users consume less than what is medically recommended, what Baicker and colleagues call *behavioral hazard*. These consumer behaviors are leveraged in interventions that seek to set out-of-pocket costs optimally, so users are motivated to use the recommended amounts. Value-based insurance design (VBID) programs go farther by targeting the reductions in cost to users to those at higher risk and by reducing the relative cost to users of alternatives that are more effective or as effective but cheaper. Plans implement these interventions because the increased high-value health-related consumption averts more serious and expensive-to-treat diseases and conditions. Baicker and colleagues did not include patient income as an explanatory variable in their models. However, a recent study (15) and earlier findings from the Rand Health Insurance Experiment (HIE) (16) corroborate that the response to changes in out-of-pocket cost do not vary substantially across income levels.

This economic review takes a plan perspective, in which the cost of the program would include the cost to cover what was previously paid by the user and the cost of providing the increased quantity demanded by users facing a lower price. The benefits would be averted long-term health care costs due to improved health of the insured. Additionally, because the plan has to pay for and implement these programs, the financial viability of the interventions is central to their likelihood of implementation.

Databases searched for this review were Cochrane, EBSCOhost, EMBASE, Web of Science, Gateway, MEDLINE/PubMed, and ProQuest. The search period was January 1980 to July 2015. A concurrent search was conducted for studies that provided economic information about these interventions, with the addition of specialized databases maintained in CRD York and EconLit.

Reference lists of articles reviewed, as well as lists in reviewed articles, were searched, and subject matter experts were consulted. The complete search strategy is available at www.thecommunityguide.org/cvd/supportingmaterials/index.html.

Studies were included as a source of evidence for this review if they 1) were published in English; 2) were conducted in a high-income country as classified by The World Bank (17); 3) had the following study designs: randomized controlled trials (RCTs), a design with a concurrent comparison group, uncontrolled before–after, or post-only with a comparison group; 4) reported at least one blood pressure or lipid outcome; 5) had 50% or more of the study population with dyslipidemia or primary hypertension, regardless of other CVD risk factors (eg, diabetes); and 6) had less than 50% of the study population with a history of cardiovascular events.

Each study that met the inclusion criteria was screened by 2 reviewers using standard Community Guide criteria; study data were abstracted and assessed for suitability of design using the standard abstraction form (www.thecommunityguide.org/methods/abstractionform.pdf) (13). Data were collected on outcomes of interest, participant demographics, intervention characteristics, applicability/generalizability, additional benefits, potential harms, considerations for implementation, and evidence gaps. Disagreements between reviewers were reconciled by consensus.

Suitability of study design was classified as greatest, moderate, or least. Studies that collected data on intervention and comparison populations prospectively were classified as having greatest suitability of design. Those that collected data retrospectively or lacked a comparison group but conducted multiple pre–post measurements had moderate design suitability. Studies without a comparison group providing before and after measurements had least suitable designs.

Threats to validity, such as poor descriptions of the intervention, population, sampling frame, and inclusion/exclusion criteria; poor measurement of exposure or outcome; poor reporting of appropriate analytic methods; loss to follow-up; or intervention and comparison groups not being comparable at baseline were used to characterize studies as having good, fair, or limited quality of execution. Studies with limited quality of execution were excluded from analysis.

Medication adherence was assessed by using 2 outcomes: change in proportion of patients adhering to prescribed medications for hypertension or hyperlipidemia, measured by using medication possession ratio, and change in proportion of patients achieving a high level of adherence, typically those who refill and possess medication 80% of the time (18).

The minimum requirements for this outcome were established standards for blood pressure control as of 2012 (<140/90 mm Hg [systolic/diastolic] or <130/80 mm Hg for people with diabetes) (19). For each study, using data from the last available point in an ongoing intervention, the team calculated the absolute (percentage point) change in the proportion of patients receiving ROPC who achieved blood pressure control compared with a reference group or pre-ROPC value.

For each study, the effect estimate for change in mean systolic blood pressure (SBP), diastolic blood pressure (DBP), low-density lipoprotein (LDL) cholesterol, triglycerides, and total cholesterol was calculated by using the last available point in an ongoing intervention for patients receiving ROPC compared with a reference group or pre-ROPC value. Outcomes pertaining to illness and death were collected and analyzed when reported.

Because study designs and reporting of outcomes for medication adherence were heterogeneous, conducting a meta-analysis was not appropriate. Therefore, descriptive statistics that facilitated simple and concise summaries of study result distribution were used for primary and secondary outcomes.

Individual effect estimates were calculated for each outcome. Percentage point (PP) changes were calculated for medication adherence and for the following at goal: blood pressure, LDL cholesterol, total cholesterol, and hemoglobin A1c (A1c). Absolute mean differences were calculated for change in mean SBP, DBP, total cholesterol, LDL cholesterol, triglycerides, A1c, and fasting blood glucose. For overall summary measures, the median of effect estimates from individual studies and interquartile intervals (IQIs) were reported for each outcome based on suitability of study design. IQIs were calculated when the body of evidence included more than 4 studies; otherwise, ranges were reported.

## Results

**Figure 2:**
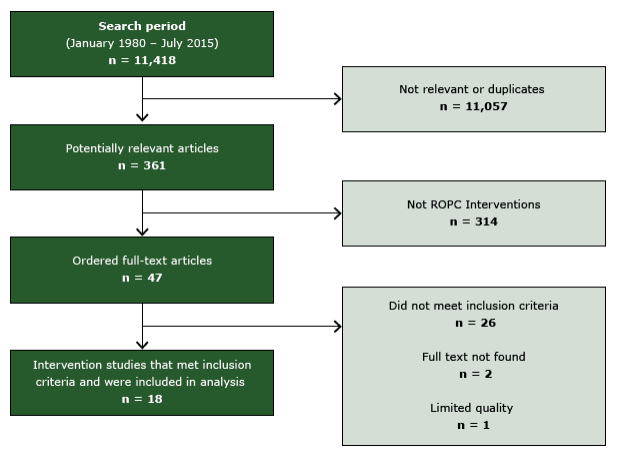
Flow diagram, showing number of studies identified, reviewed in full text, reasons for exclusion, and total number of included studies. Abbreviation: ROPC, reducing patient out-of-pocket costs.

After screening 11,418 titles and abstracts, we selected 47 studies for full-text review; 18 studies met inclusion criteria (20–37) (Figure 2). One study was excluded for limited quality of execution (38) and 3 provided information on an already-included study (39–41). Details of the included studies are available at www.thecommunityguide.org/cvd/supportingmaterials/IS-ROPC.html.

Of the 18 included studies (20–37), 15 were conducted in the United States (20,21,23–25,27–31,33–37), one in Israel (26), one in Italy (22), and one in Australia (32). All studies evaluated programs or policies that implemented ROPC for medications to treat patients with hypertension, hyperlipidemia, or both. Moreover, 7 of the 18 included studies used a VBID plan (24,25,27,28,33,34,37) and 3 studies used pharmaceutical medication assistance programs (PMAP) programs to procure medications for indigent patients (29,35,36). Although most studies also implemented medication counseling or patient education, they did not report ROPC for these services. Seven studies used a team-based care approach combined with medication counseling (21,23,24,29,30,35,36); 7 studies also evaluated interventions that eliminated medication costs but did not specify if medications were generic or brand name (20–22,26,29,30,35). Nine studies provided generic medications free of charge and brand name medications at reduced cost (23–25,27,32–34,36,37), and one evaluated reduced coinsurance (cost for covered benefits the insured pays after the deductible has been paid) (28).

Most studies reported implementing ROPC for medications with one or more health care intervention components, such as medication counseling. Two studies (20,26) did not report implementing specific health care interventions — such as medication counseling — with ROPC, although it is unlikely that patients in these studies received ROPC for medication without receiving a new or existing health care intervention (eg, patient education).

Study populations primarily included working-age adults (median age 54.7 y) with more women than men participating (Table 1). Studies included diverse racial/ethnic groups, which were predominantly white in 3 studies (20,23,37), African American in 2 (21,30), and Hispanic in one (29). Patients in 12 studies were fully insured (20,22–25,27,28,31–34,37); patients in 7 of those studies were fully insured under a VBID plan (24,25,27,28,33,34,37). Six studies included mostly uninsured or underinsured low-income patients (21,26,29,30,35,36).

**Table 1:** Studies (N = 18) Reporting Population Characteristics for Interventions That Reduce Patient Out-of-Pocket Costs for Medications to Treat Hypertension and Hyperlipidemia, January 1980 to July 2015

Characteristic	Category	Number of Studies Reporting Characteristic (% of total)^a^
Age, y	Adult (18–64)	10 (56)
	Older adults (>64)	4 (22)
Sex	Majority female	12 (67)
	Majority male	3 (17)
Race/Ethnicity	Majority white	3 (23)
	Majority African American	2 (15)
	Majority Hispanic	1 (8)
Income level	Majority low-income	6 (46)
Type of benefit design^b^	Fully insured	12 (67)
	Fully insured under VBID	7 (39)
	Underinsured/uninsured	6 (46)

Abbreviations: VBID, value-based insurance design.

a Total number of studies (and proportion) that reported specific demographic characteristic. Because some studies provide no information on variable of interests, totals do not add up to 100%.

b Categories are not mutually exclusive.

Seven studies evaluated the effectiveness of ROPC on patients’ medication adherence, measured as the percentage of time a patient is in possession of a prescribed medication (24,25,27,28,33,34,37) (Table 2). All 7 studies evaluated patients in VBID plans. Six studies measured overall adherence for 15 blood pressure and lipid medications, and found adherence rates increased by a median of 3.0 percentage points (IQI = 2.3, 4.5 PPs). The remaining VBID study reported a 5.1 percentage point increase in the proportion of patients achieving 80% adherence to blood pressure medications (28).

**Table 2:** Effects of Reducing Patient Out-of-Pocket Costs for Medications to Treat Hypertension and Hyperlipidemia on Medication Adherence, Blood Pressure Outcomes, and Lipid Outcomes in 9 Studies Published From January 1980 Through July 2015

Review Outcome	Effectiveness Measurements	Suitability of Study Design (No. of Studies)	Summary Estimates
Medication adherence	PP change in patient adherence rates for blood pressure and cholesterol medications	Greatest (6 studies with 15 study medications) (24,25,27,33,34,37)	Median: increase of 3.0 PPs (IQI, 2.3 to 4.5 PPs)
	PP change in proportion of patients achieving 80% adherence	Greatest (1 study) (28)	Increase of 5.1 PPs
Blood pressure (BP) at goal	PP change in proportion of patients with controlled BP	Greatest or moderate (3 studies) (30,31,36)	Median: increase of 6.0 PPs (range,−8.2 to 17 PPs)
		Least (4 studies) (20,21,23,37)	Median: increase of 30.1 PPs (IQI, 20.3 to 46.5 PPs)
Systolic blood pressure (SBP)	Change in mean SBP (mm Hg)	Greatest or moderate (4 studies (29–31,36)	Median: decrease of 5.9 mm Hg (range, –10.7 to 3.83 mm Hg)
		Least (6 studies) (20,21,23,26,35,37)	Median: decrease of 8.7 mm Hg (IQI, −14.5 to –5.45 mm Hg)
Diastolic blood pressure (DBP)	Change in mean DBP (mm Hg)	Greatest or moderate (4 studies) (29–31,36)	Median: decrease of 3.75 mm Hg (range, –6.1 to –2.1 mm Hg)
		Least (6 studies) (20,21,23,26,35,37)	Median: decrease of 4.5 mm Hg (IQI, –7.8 to –3.8 mm Hg)
Low-density lipoprotein (LDL) cholesterol	Change in mean LDL cholesterol (mg/dL)	Greatest or moderate (3 studies) (29,36,37)	Median: reduction of 14 mg/dL (range, –16 to –6.9 mg/dL)
		Least: (3 studies) (23,26,35)	Median: reduction of 14 mg/dL (IQI, –18.9 to 10.9 mg/dL)
	PP change in proportion of patients achieving LDL cholesterol goal	Greatest or moderate (2 studies) (36,37)	Median: increase of 18.5 PPs (range, 13 to 24 PPs)
		Least (1 study) (23)	Increase of 10 PPs
Triglycerides (TG)	Change in mean TG (mg/dL)	Greatest or moderate (2 studies) (29,37)	Median: reduction of 11.4 mg/dL (range, –13.0 to –9.8 mg/dL)
		Least (2 studies) (23,35)	Median: reduction of 31.7 mg/dL (range, –38.4 to 25.0 mg/dL)
Total cholesterol (TC)	Change in mean TC (mg/dL)	Greatest (1 study) (29)	Reduction of 15 mg/dL
		Least (1 study) (35)	Reduction of 25 mg/dL
	PP change in proportion of patients achieving TC goal	Greatest (1 study) (30)	Decrease of 7.0 PPs

Abbreviations: IQI, interquartile interval; PP, percentage points.

Two studies examined adherence in non-VBID populations using different measurements for adherence. One study — in a non-VBID population — reported that medication adherence increased by 21.4 percentage points among those with low baseline adherence (<55%) but decreased by 2.2 percentage points among those with high baseline adherence (22). Another study, conducted in Australia, found that patients who did not hold a concession card, which reduced out-of-pocket costs, were 1.63 times more likely to be nonadherent to statin therapy (32).

The results for blood pressure (Table 2) are based on suitability of design. For studies reporting proportion of patients with controlled blood pressure, 3 studies with greatest or moderate design suitability showed a median improvement of 6.0 percentage points (30,31,36); 4 studies with least suitable designs reported a median improvement of 30.1 percentage points (IQI = 20.3, 46.5 PPs) (20,21,23,37). For change in SBP, 4 studies with greatest or moderate design suitability reported a median reduction of 5.9 mm Hg (29–31,36). Six studies with least suitable designs had a median reduction of 8.7 mm Hg (IQI = –14.5, –5.45 mm Hg) (20,21,23,26,35,37). Similarly, for change in DBP, 4 studies with greatest or moderate design suitability reported a median reduction of 3.75 mm Hg (29–31,36); 6 studies with least suitable designs reported a median reduction of 4.5 mm Hg (IQI = –7.8, –3.8 mm Hg) (20,21,23,26,35,37).

**Blood pressure outcomes among low-income populations.** Six studies reported blood pressure outcomes among majority low-income patient populations; 3 had greatest or moderate suitability of design (29,30,36) and 3 had least suitable study designs (21,26,35). Two of 3 studies with greatest or moderate suitability reported proportion of patients with blood pressure controlled; the median improvement was 4.4 percentage points (range = –8.2, 17.0 PPs) (30,36). Only one study with least suitable design reported blood pressure control, with an overall improvement of 51 percentage points (21). All 6 studies reported mean changes in SBP and DBP. For 3 studies of greatest and moderate suitability (29,30,36), the median reductions were 10 mm Hg (range = –10.9, 5.7 mm Hg) and 5 mm Hg (range = –6.4, –2.5 mm Hg), respectively; the 3 studies of least suitable design reported median reductions of 8 mm Hg (range = –24.8, 2.0 mm Hg) and 6 mm Hg (range = –13.1, –3.2 mm Hg), respectively (21,26,35).

**Value-based Insurance Design.** Only one study reported clinical outcomes for fully insured patients with a VBID plan (37). That study reported an increase of 18.0 percentage points for proportion of patients with blood pressure controlled. Changes in mean SBP and DBP were mean reductions of 6.6 mm Hg and 4.2 mm Hg, respectively.

**Pharmaceutical medication assistance programs.** Three studies focused on reported outcomes for blood pressure in PMAP populations (29,35,36). For proportion of patients with blood pressure at goal, one study reported an unfavorable decrease of 8.2 percentage points (36). All 3 studies reported outcomes for SBP and DBP. Two studies with greatest and moderate suitability of design reported median reductions of 2.15 mm Hg and 3.75 mm Hg, for SBP and DBP respectively (29,36); one study with least suitable design reported mean reductions of 2.0 mm Hg and 6.0 mm Hg, respectively (35).

Results for 6 studies (Table 2) evaluated ROPC effects on lipid outcomes in target populations, including VBID and PMAP (23,26,29,35–37). ROPC interventions were effective in improving change in total cholesterol, LDL cholesterol, and triglycerides. Favorable results were also reported for proportion of patients with LDL cholesterol at goal, although one study reported unfavorable results for total cholesterol at goal (30).

## Additional Evidence

**Illness and death outcomes.** Two studies assessed ROPC effects on illness or death (22,23). One employer-initiated study reported significant reductions in rate of myocardial infarction (odds ratio [OR] = 0.24; 95% confidence interval [CI], 0.098–0.594) and any CVD events (OR = 0.47; 95% CI, 0.328–0.671) during the intervention period compared with the historical period (23). The other study reported that among patients with low baseline adherence (<55%), hospitalization rates decreased from 7.9% to 7.0% and mortality rates decreased from 3.4% to 3.2%; both reductions were significant at *P* < .05 (22).

The economic search identified 9 studies for inclusion in the economic review (23,26,28,33,34,37,40,42,43), of which 7 were evaluations of VBID programs (28,33,34,37,40,42,43). All the studies evaluated interventions that reduced the cost of medications (Table 3). Interventions in addition to ROPC were reported in 5 of the studies, 2 with team-based care (23,37) and 3 with disease or lifestyle management offered in addition to VBID benefits (28,34,43). Only one study targeted a low-income population (26). No studies reported cost-effectiveness of the intervention. All monetary values reported are in 2014 US dollars, using the Consumer Price Index from the Bureau of Labor Statistics (44) and purchasing power parities from the World Bank for conversions (45).

**Table 3:** Intervention Cost, Health Care Cost, and Net Benefit of Reducing Patient Out-of-Pocket Costs (ROPC) for Medications to Treat Hypertension and Hyperlipidemia in 9 Studies Published From January 1980 Through July 2015

Study	Size of Intervention Group, Length of Follow-up	VBID	Cost of ROPC for Medications Per Patient Per Year	Cost of Other Intervention Components Per Patient Per Year	Health Care Cost Per Patient Per Year (Components)	Net Benefit Per Patient Per Year
Bunting et al 2008 (23)	N = 620, 5 y	No	$676	TBC: NR	−$759 (OP, IP, ER)	NR
Elhayany^a^^,^ b and Vinker 2011 (26)	N = 938, 1 y	No	$642	NA	NR	NR
Wertz et al 2012 (37)	N = 307, 14 mos	Yes	$45	TBC: $541	−$249 (OP, IP, ER)	−$337
Gibson et al^c^ 2011 (28)	N = 2,873, 2 y	Yes	$78	Disease management: NR	−$2,417 Yr 1, −$4,240 Yr 2 (OP, IP)^d^	NR
Kelly et al 2009 (40)	N = 1,550, 2 y	Yes	$205	NA	−$114 (OP, IP, ER)	−$90
Chernew et al^b^ 2010 (42)	NR, NR	Yes	$116	NA	NR	Modeled assumptions suggest that VBID is cost-neutral
Choudhry et al 2012 (43)	N = 2,051, 1 y	Yes	$16	Disease management: NR	$14 (OP, IP, ER, Long-term care)^d^	NR
Maciejewski et al 2014 (33)	N = 750,000, 1 y	Yes	$153–$190	NA	Patients with high blood pressure only: −$158 in year 1 and −$74 in year 2	$0, cost-neutral
					Patients with high blood pressure and hyperlipidemia: −$160 in year 1 and −$116 in year 2 (OP, IP, ER)^d^	
Musich et al 2015 (34)	N = 2,674, 2 y	Yes	Patients with high blood pressure: $491	Disease/lifestyle management coaching: NR	Patients with high blood pressure: $376 (OP, IP)^d^	NR

Abbreviations: ER, emergency department; IP, inpatient; NA, not applicable; NR, not reported; OP, outpatient; ROPC, reduced out-of-pocket costs; TBC, team-based care; VBID, value-based insurance design.

a Study conducted in Israel; all other studies were conducted in the United States.

b Study targeted low-income patients.

c Includes blood pressure, cholesterol, diabetes, and asthma medications covered under VBID.

d Compared with control.

The intervention cost per person per year of increased pharmacy spending by plans was provided by all 9 studies, with median = $172 (IQI: $70 to $529, n = 10). The higher estimates included blood pressure-lowering and diabetes medications. Of the 5 studies that had interventions in addition to ROPC, only one also provided the cost of the additional team-based care component (37).

Seven studies estimated change in health care cost, with median = –$127 (IQI: –$632 to –$18, n = 8) (23,28,33,34,37,40,43), where all but 2 included interventions in addition to ROPC (33,40). In the context of these multiple intervention studies, the observed effects on health outcomes and health care cost result from the combined interventions rather than ROPC alone. The follow-up period for these studies ranged from 1 year to 5 years, with most lasting from 1 to 2 years, inclusive.

The elasticity of medication adherence could not be calculated for the included studies because either the information was not available or the change in adherence was due to interventions in addition to ROPC.

Of 3 studies that reported sufficient information to compute net benefits, 2 found the cost of intervention exceeded averted health care costs by $337 (37) and $90 (40) per patient per year, and the third found the intervention to be cost-neutral (33). Hence, the evidence for net benefit is mixed and, in particular, the 2 studies that were not combined interventions indicate VBID was cost-neutral in one instance (33) and cost-increasing in another (40).

## Discussion

Findings from this review are applicable to the US health care system and working-age adults. Although patients from both sexes and diverse racial and ethnic groups were well represented, evidence from this review indicates that ROPC is especially beneficial for low-income patients. Moreover, ROPC interventions are applicable to diverse policy and program implementers, such as employers and government agencies.

Coordination of ROPC with additional interventions (eg, medication counseling) may increase opportunities for patient–provider interaction on treatment issues (eg, medication side effects). Neither the included studies nor the broader literature identified any harms to patients from these interventions.

According to Community Guide rules of evidence (12), there is strong evidence that ROPC for medications to treat hypertension and hyperlipidemia is effective in 1) improving medication adherence and 2) improving blood pressure and cholesterol outcomes, when implemented in combination with evidence-based health care interventions such as team-based care with medication counseling. However, an overall conclusion cannot be reached regarding the economics of the intervention from the small body of inconsistent evidence on net benefits.

This review examined effectiveness of ROPC for medications to treat hypertension and hyperlipidemia. Findings are consistent with those from an Agency for Healthcare Research and Quality (AHRQ) systematic review of interventions to improve medication adherence for chronic diseases (46). The broader review identified 5 studies that examined ROPC for patients with CVD. Because our review focused on CVD prevention (ie, hypertension, hyperlipidemia), it excluded some studies included in the AHRQ review; nonetheless, both reviews reached a similar conclusion despite having different inclusion criteria. To the authors’ knowledge, this is the only systematic review focused on CVD prevention to examine the relationship between cost-sharing and medication adherence in patients with hypertension and hyperlipidemia.

The impact of patient cost-sharing was first assessed in the groundbreaking study, the RAND HIE, conducted during 1971–1982 (16). Although the HIE results have influenced many insurance plans’ cost-sharing policies for office visits for preventive services, evidence for cost-sharing on prescription drug plans is still sparse, especially for chronic disease management. The body of evidence in this review — albeit small — indicates that progress has been made in evaluating the impact of cost-sharing on medication adherence in people with chronic diseases. Of 18 studies included in this review, only 2 were published before 2000 (20,31); this trend appears to be the same for the 5 ROPC studies in the AHRQ review (46). The team postulates that the lack of studies before 2000 could be due to several factors: the focus of this review on CVD prevention; financing model of prescription medications in earlier years (eg, cash only); changes in insurance designs (eg, medical insurance plus prescription drug plans); or possible publication bias during 1980–2000.

Implementation of ROPC interventions primarily has implications for health policy decision makers considering changes to health insurance and prescription drug plans. Several opportunities exist for innovative application of ROPC programs and policies. Ideally, ROPC for hypertension and hyperlipidemia treatment will be implemented along with other CVD preventive services. A comprehensive approach might coordinate ROPC for medications to treat hypertension and hyperlipidemia with ROPC for evidence-based treatments of tobacco cessation (47) and management of patients with diabetes. Additionally, prescribing providers or others in the health care system can be advocates for their patients by 1) actively asking patients about their ability to pay for medications and 2) familiarizing themselves with medications covered by patients’ health insurance plans with no or low out-of-pocket costs to patients. A more complete discussion of considerations for implementation is available (48).

This review has several limitations. The quality of included studies varies, with only 2 RCTs. Because ROPC interventions tend to be policy-based, a broad approach was taken to include observational study designs, thus allowing assessment of these interventions in real-world, practice-based settings. However, because most included studies were observational, the validity of this review may be threatened by biases associated with observational studies (eg, confounding, selection bias), leading to intervention effects in a favorable direction. Furthermore, because of heterogeneity in study designs and reporting of outcomes for medication adherence, a meta-analysis was not conducted; hence, descriptive statistics were used to summarize findings of this review. Visual inspection of funnel plots examining the relationship between effect size and sample size indicated possible publication bias for clinical and medication adherence outcomes, with most outcomes reporting favorable results.

Future studies should report whether health improvements are directly associated with incremental reductions in patient out-of-pocket costs and describe ROPC effects on both medication adherence and clinical outcomes. Furthermore, natural experiments are needed to comparatively evaluate the impact ROPC interventions have on medication adherence, clinical outcomes, and health behavior outcomes. Lack of reporting also precluded the team from evaluating how access to medication influenced medication adherence, although it can be assumed that PMAP made brand-name medications readily accessible to indigent patients. Future research should also investigate the impact of cost-sharing and medication adherence on high-income socioeconomic groups, where the marginal cost-share difference is expected to be a smaller proportion of total income.

The absence of cost-effectiveness evaluations of these interventions needs to be addressed in future research. Furthermore, economic evaluations should provide the cost of implementing both the ROPC and the additional intervention when ROPC is combined with interventions such as team-based care.
